# A laryngeal presentation of Churg--Strauss syndrome in childhood

**DOI:** 10.4103/0256-4947.51807

**Published:** 2009

**Authors:** Ahmed Y. Al-Ammar, Subhan S. Yasin, Saleh Zaid Al-Muhsen, Muslim M. Al-Saadi, Mohammad O. Al-Sohaibani

**Affiliations:** a Department of Otolaryngology, Head and Neck Surgery, King Abdulaziz University Hospital, Riyadh, Saudi Arabia; b Department of Pediatrics, King Abdulaziz University Hospital, Riyadh, Saudi Arabia; c Department of Pathology, King Abdulaziz University Hospital, Riyadh, Saudi Arabia

## Abstract

A 10- year-old female, known to have bronchial asthma, presented with an unusual laryngeal lesion, eventually diagnosed as Churg-Strauss syndrome (CSS). She was referred to our hospital with history of recurrent stridor. On endoscopyhe, the larynx showed signs similar to recurrent respiratory papillomatosis (RRP). CSS is a systemic disorder and is now defined as one of the ANCA (antineutrophil cytoplasmic antibodies)-associated vasculitides. CSS is a systemic disease that may involve unusual sites like the larynx. Such an unusual presenatation of CSS should be kept in mind, especially in patients with history of asthma.

Churg-Strauss syndrome (CSS) is a systemic disease characterized by asthma, blood and tissue eosinophilia, and necrotizing vasculitis with extravascular eosinophilic granulomas.^1^ It is defined as one of the ANCA (antineutrophil cytoplasmic autoantibody)-associated vasculitides.^2^ Despite recent interests, it is still a rare disease with a poorly understood pathogenesis. The prevalence of CSS was recently estimated at 10.7 per million adults in France.^3^ Ear, Nose nose and throat involvement is a common finding, usually manifesting as allergic rhinitis and chronic rhinosinusitis with or without nasal polyposis.^4^ Other rare symptoms like otitis media, facial palsy and vocal cord paralysis are also seen. In this report, we describe a unique case of CSS which presented with laryngeal mass, stridor and findings resembling recurrent respiratory papillomatosis (RRP).

## CASE

A 10-year-old girl was referred to our institution, King Abdulaziz University Hospital, Riyadh, Saudi Arabia, for the management of stridor. According to the referral history, the patient was known to have bronchial asthma and used medication as needed and presented initially with difficulty in breathing at the age of 9 years. She apparently had no other medical history and was healthy prior to the first presentation. On endoscopy a growth had been seen in the larynx similar to polyposis of the larynx, and she was diagnosed as a case of RRP. That mass was excised. She again developed the same symptoms within 7 months and was operated on for another mass. She was referred for further management because of the recurrence of her symptoms.

At the time of presentation to our institute 2 months after the last surgery she had biphasic stridor, with minimal signs of respiratory distress and so was admitted for a work-up. A general examination showed that her weight was 23 kg and stature was 120cm, both below the fifth percentile, and systemic examination was unremarkable. A near-nose-throat examination was unremarkable except that on fiberoptic endoscopy, the larynx showed a papillomatous growth involving both the true vocal cords and interarytenoid area. She had a narrow airway. Her vocal cords were mobile. Her blood work showed WBC count of 12.9×10^9^/L, with an eosinophil count of 60%. The erythrocyte sedimentation rate was 54 mm/hr. Due to her recurrent attacks of bronchial asthma associated with chest infections, poor weight gain and non-resolving chest symptoms and a possible history of exposure to tuberculosis in the family, a purified protein derivative test was done and the result was negative. Serology for human papilloma virus 6 and 11 was negative. c-ANCA tested positive with a titer of 28 c-ANCA tested positive with a titer of 32 AU (more than 15 AU is considered positive, normal up to 20 AU), levels were measured using a commercially available direct PR3-ANCA ELISA kit (Euro-diagnostica, Malmo, Sweden).

**Figure 1:**
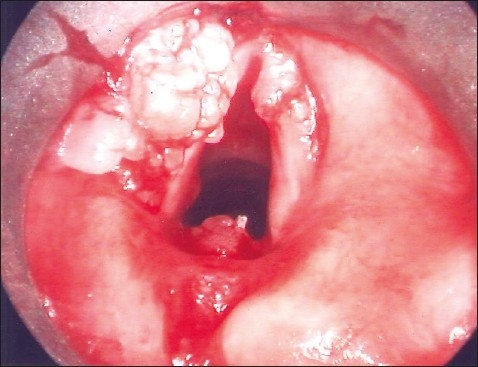
The lesion involving both vocal cords and interarytenoid area.

**Figure 2:**
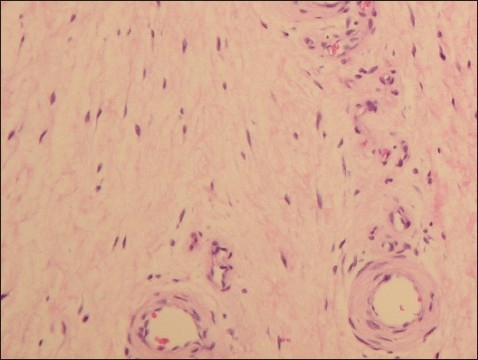
Section shows multiple vessels with evidence of vasculitis. The vessel walls show fibrin deposits. Perivascular area shows number of eosinophils, plasma cells and macrophages. (Hematoxylin and eosin stain original magnification ×128).

Direct laryngoscopy confirmed involvement of the glottic region with a papillomatous lesion bilaterally with extension into the subglottic region mainly on the left side (Figure 1). Excision of the lesion was done using CO_2_ laser and the growth was sent for histopathology, which showed multiple vessels with evidence of vasculitis and fibrin deposits in the walls (Figure 2). A number of eosinophils, plasma cells and macrophages were seen in the perivascular area. The diagnosis of CSS was made based on both histopathological and serological findings along with suggestive clinical presentation.

**Figure 3:**
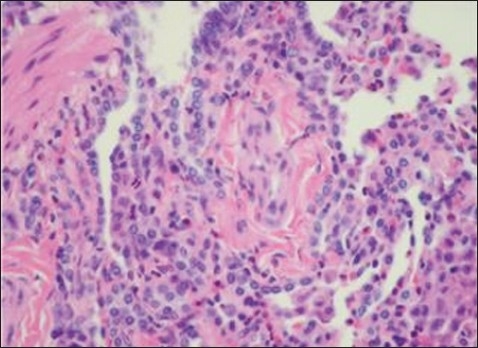
Section shows lung tissue with parenchymal fibrosis, lymphocytic, plasma cells and eosinophilic infiltration along with fibrinoid thickening of medium-sized blood vessels. (Hematoxylin and eosin stain original magnification ×320).

Within the next two months the patient had recurrence of symptoms and was again admitted with stridor. Along with stridor, she had developed a mass in the right vestibule of the nose. Flexible fiberoptic examination showed papillomatous growth involving the true vocal cord on the left side, the anterior commissure, the interarytenoid region, the subglottic region and the dorsum of the posterior third of tongue. Her vocal cords were mobile. Another surgical intervention was made to clear the airway. Excisional biopsies of the nasal mass and tongue base mass were done during the same intervention. A CT scan of the chest done postoperatively showed chronic inflammatory airway disease with a small rounded lesion in the upper lobe of the right lung. A repeat lung biopsy, which was a firm piece of tissue measuring 1×0.7×0.2 cm whose sections revealed lung tissue with parenchymal fibrosis, lymphocytic, plasma cells and eosinophilic infiltration along with fibrinoid thickening of medium-sized blood vessels and pulmonary tissue with multiple vessels showing evidence of vasculitis (Figure 3) The vessel wall showed fibrin deposits. Perivascular area showed a number of eosinophils with plasma cells and macrophages. The lung biopsy was consistent with the same pathology (CSS), as were tongue and nasal biopsies. The specimens also underwent Gomori methenamine silver and Steiner stains and was negative. Immunostains were not suggestive and culture showed no growth of micro-organisms.

At the time of the latest presentation the child was started on prednisone at a dose of 1 mg/kg/day for 6 months and this dose was tapered down over 4 month. The child was under regular follow up both by the pediatrician and our team with no further airway compromise. Her last follow-up visit was 4 years after the last intervention, when flexible fiberoptic examination of her larynx showed a clear airway. She had, however, before the last follow-up been to the emergency department from time to time for acute exacerbation of bronchial asthma. During the course of follow-up there was no sign suggesting neurological or cardiac involvement.

## DISCUSSION

CSS is a systemic disease also referred to as allergic granulomatosis, which was first described in 1951 by Jacob Churg and Lotte Strauss as a syndrome consisting of “asthma, eosinophilia, fever, and accompanying vasculitis of various organ systems”.^1^ Other types of systemic vasculitides are the primary vasculitis like polyarteritis nodosa and Wegener's granulomatosis and secondary vasculitis which are complications of a connective tissue disorder like rheumatoid arthritis, infection, medication or malignancy. CSS shares many of the clinical and pathological features of polyarteritis nodosa (“PAN”, another type of vasculitis). Churg and Strauss discovered that the presence of granulomas as well as the abundance of eosinophils distinguished this disease from PAN. The American College of Rheumatology (ACR) has established criteria that must be fulfilled to classify a patient as having CSS.^5^ These criteria were intended to distinguish CSS from other forms of vasculitis. To be classified as having CSS, a patient should have at least 4 of the 6 ACR criteria: 1) asthma, 2) eosinophilia (> 10% on differential WBC count), 3) mononeuropathy, 4) transient pulmonary infiltrates on chest X-rays, 5) paranasal sinus abnormalities and 6) a biopsy containing a blood vessel with extravascular eosinophils. The presence of 4 or more of these 6 criteria yields a sensitivity of 85% and a specificity of 99.7%.^5^

Our patient clearly fulfilled five of six criteria outlined by the ACR. Interestingly, she is one of the few known cases of CSS primarily presenting with laryngeal symptoms and with laryngeal pathology. In a previous case reported by Mazzantini et al the patient's first clinical manifestation was a persistent dysphonia.^6^ A videolaryn gostroboscopic examination revealed paresis of the right vocal fold with a reduction in adduction together with incomplete glottal closure. Laryngeal electromyography revealed neurogenic damage of the right thyroarytenoid and crycoarytenoid muscles. In the reported case there was only neurological involvement of the larynx, but in our case the larynx was involved by a mass lesion, which is why our case is unique in presentation. To our knowledge, this is the first case reported in the literature with such laryngeal findings. There are numerous other studies of CSS presenting with gastrointestinal symptoms or with nasal symptoms, particularly allergic rhinitis and nasal polyposis,^4^ and occurrence in children is infrequent.^7^ Other symptoms like otitis media, facial palsy and vocal cord palsy are also seen, but rarely. Subglottic involvement of the larynx with vasculitis lesions in cases of Wegener's granulomatosis is reported, but is a rare finding.^8^,^9^ Grans et al suggested that the presence of autoantibodies along with subglottic stenosis places such patients within the spectrum of necrotizing (granulomatous) vasculitis.^10^ Other clinical studies^11^ found that the clinical characteristics of patients with CCS varied according to their ANCA status: cardiomyopathy is predominant in ANCA-negative patients while necrotizing glomerulonephritis is more often observed in ANCA-positive patients. These histologically documented findings suggest the existence of different CCS subtypes, characterized by a predominance of distinct pathogenetic mechanisms.

A variety of non-invasive imaging techniques is now becoming available to investigate patients with vasculitis.^12^ These include ultrasonography, MRI coupled with angiographic sequences, positron emission tomography, single photon emission computed tomography (SPECT). Their role is being evaluated and their characteristics exploited to address issues specific to each vasculitis

In conclusion, CSS is a systemic disorder and are now defined as one of the ANCA-associated vasculitites. Despite recent interest, it is still a rare disease with a poorly understood pathogenesis. This paper describes a unique case of CSS, which presented with laryngeal mass, stridor and findings resembling RRP. CSS is a systemic disease that may involve unusual sites like the larynx. Such an unusual presenatation of CSS should be kept in mind, especially in patients with history of asthma.
